# Characteristics and Key Features of Antimicrobial Materials and Associated Mechanisms for Diverse Applications

**DOI:** 10.3390/molecules28248041

**Published:** 2023-12-11

**Authors:** Aaruci Agarwalla, Waleed Ahmed, Ali H. Al-Marzouqi, Tahir A. Rizvi, Mushtaq Khan, Essam Zaneldin

**Affiliations:** 1 Department of Chemical and Petroleum Engineering, College of Engineering, United Arab Emirates University, Al Ain P.O. Box 15551, United Arab Emirates; 700043169@uaeu.ac.ae (A.A.);; 2 Engineering Requirements Unit, College of Engineering, United Arab Emirates University, Al Ain P.O. Box 15551, United Arab Emirates; 3 Department of Microbiology & Immunology, College of Medicine & Health Sciences, United Arab Emirates University, Al Ain P.O. Box 15551, United Arab Emirates; 4 Zayed Center for Health Sciences, United Arab Emirates University, Al Ain P.O. Box 15551, United Arab Emirates; 5 Department of Civil and Environmental Engineering, College of Engineering, United Arab Emirates University, Al Ain P.O. Box 15551, United Arab Emirates; essamz@uaeu.ac.ae

**Keywords:** antimicrobial material, 3D printing, antibacterial, antiviral, antimicrobial mechanism, metals, polymers, ceramics, applications

## Abstract

Since the Fourth Industrial Revolution, three-dimensional (3D) printing has become a game changer in manufacturing, particularly in bioengineering, integrating complex medical devices and tools with high precision, short operation times, and low cost. Antimicrobial materials are a promising alternative for combating the emergence of unforeseen illnesses and device-related infections. Natural antimicrobial materials, surface-treated biomaterials, and biomaterials incorporated with antimicrobial materials are extensively used to develop 3D-printed products. This review discusses the antimicrobial mechanisms of different materials by providing examples of the most commonly used antimicrobial materials in bioengineering and brief descriptions of their properties and biomedical applications. This review will help researchers to choose suitable antimicrobial agents for developing high-efficiency biomaterials for potential applications in medical devices, packaging materials, biomedical applications, and many more.

## 1. Introduction

Due to Industrial Revolution (IR) 4.0, the manufacturing environment has rapidly shifted from traditional strategies to emerging alternatives with intelligent production systems and cutting-edge technologies [1]. Additive manufacturing (AM) played a crucial role in IR 4.0 because of its exceptional potential to offer a green manufacturing alternative, with advanced design flexibility, which outperforms conventional manufacturing methods, and its observable effectiveness in performance, precision, time, and cost. [1]

The application of three-dimensional (3D) printing, a type of AM, has enabled the manufacturing of various devices with potential applications in several fields, including mechanical, aerospace, medical, tissue engineering, and bioengineering fields [1]. AM methodologies enable the construction of structures with complex geometrical cues and high accuracy, closely similar to biological tissues. There is an increasing demand for novel manufacturing methods that can address the issue of tissue and organ shortages. These technologies should meet the immunological requirements of implanted devices while also addressing the growing demand for, and applications of, tissue engineering, antimicrobial devices, and regenerative medicine. Various biomaterial-based medical implants and devices are being used to increase individuals’ life expectancy and quality of life [2].

Nonetheless, material contamination by various microorganisms can impair the quality of medical equipment and healthcare goods, being a key constraint in using biomaterials for biomedical applications [2]. Since their first appearance millions of years ago, microorganisms, including bacteria, fungi, viruses, algae, and other living things, have been affecting every element of existence. Microbial infections can directly or indirectly cause many human diseases [2]. Microbial surface contamination, which includes adherence of bacteria to surfaces, their presence on these surfaces for extended periods, and surface colonization through microbes, harms human health and society [3]. Nosocomial and community-acquired infections are mainly transmitted by touching infected surfaces [2]. In the United States, more than 90,000 individuals die every year because of nosocomial infections [2]. Antibiotic-resistant microbes and biofilm-associated infections (microorganism agglomerates adhered to the surface) further deteriorate the condition of individuals with nosocomial and community-acquired infections [2].

Infectious diseases caused by biofilm-producing microbes account for more than 80% of microbial infections in the body, increasing patient morbidity and medical costs [3]. Because biofilms cannot be accessed easily by antibacterial agents and the human immune system, they serve as a stockpile of bacteria, which then cause persistent, recurring infections throughout the body upon maturation (i.e., when microbes proliferate and produce extracellular polymers to form many differentiated layers of microbes) [3,4]. In addition, biofilms are resistant to conventional antibiotics. Consequently, bacterial infections are difficult to treat despite the abundance of potent modern antibiotic medications and antimicrobial agents [3].

With the increasing use of stents and other implants, biomedical devices have recently become essential to healthcare systems. Consequently, postsurgical bacterial infections, also known as device-related infections, have considerably increased, posing a serious health risk to patients [2]. Biofilm-associated infections play an essential role in device-related infections. The exponential increase in new antibiotic drugs in the post-antibiotic era can barely cope with the increase in bacterial resistance, posing a serious threat to human health [2,4]. More than 700,000 patients die annually because of antibiotic resistance or device-related infections [4]. As a result, antimicrobial materials have been used as feasible options for eliminating or minimizing microbial development, thus preventing nosocomial and biofilm-related diseases. These materials address the critical needs of public health systems [2].

Due to advancements in aseptic treatment, environmental sterility management, and antibiotic treatment during surgery, antimicrobial materials have become the primary solution for preventing device-related infections [4]. The infection resistance of medical equipment and tools has been enhanced by adding anti-infective bioactive features using antimicrobial biomaterials [4]. Antimicrobial materials can also be used to develop medicines that primarily aim to advance the biomedical field by curing, preventing, or lowering infections [4]. Microbial transmission and biofilm-associated diseases can be prevented by killing or inhibiting the growth of microbes through biocidal coatings, surface-bound active antimicrobials, or passive pathogen-repellent surfaces developed using nanomaterials or chemical modifications. The development of most antimicrobial coatings focuses more on their antibacterial activity than their antiviral activity. However, depending on the virus and surface types, the persistence of viable viruses on surfaces poses a threat of transmission via these surfaces, leading to the urgent need for developing strategies to prevent the growth of viruses on surfaces [5].

This review discusses strategies for fabricating materials with antimicrobial properties. Furthermore, the general antimicrobial mechanisms of metal and metal nanoparticles have been described, examples of metal and metal nanoparticles commonly used in biomedical science have been provided, and their antiviral properties have been briefly described. The antimicrobial mechanisms of polymers and ceramics have also been discussed and examples of them have been provided. Finally, examples of antimicrobial materials used in 3D printing have been presented.

## 2. Strategies for Fabricating Biomaterials with Antimicrobial Properties

The mechanisms of action of antimicrobial drugs should be elucidated to understand the processes involved in developing antimicrobial surfaces. Antimicrobial agents have little or no influence on host functions but specifically target key bacterial processes. Various antibacterial substances work through different pathways; understanding these pathways and the chemical composition of antimicrobial drugs is essential for determining the development of resistance. Antimicrobial substances can be broadly categorized as bacteriostatic and bactericidal [6,7]

Bacteriostatic antimicrobial agents merely prevent bacteria from growing or multiplying, giving the host’s immune system time to remove them from the body. Therefore, in this case, the immune system’s effectiveness is necessary for complete germ eradication. In contrast, bactericidal agents kill bacteria, regardless of whether the host has a functioning immune system. However, the mode of action of antimicrobial substances can still be further categorized based on the bacteria’s structure or the function they impact [6,7]. The typical modes of action are (1) prevention of cell wall synthesis, (2) impairment of ribosome activity, (3) inhibition of nucleic acid production, (4) inhibition of folate metabolism, and (5) suppression of cell membrane activity [6,7].

Numerous strategies and principles have been developed to fabricate biomaterials with antimicrobial properties [8]. Widely used approaches for decreasing the sensitivity of antimicrobial medical devices to microbial colonization and infection are summarized in this review based on two principles [4].

### 2.1. Treatment of Surfaces with Microbe-Repelling and Antiadhesive Substances

In the process of infection caused by foreign body substances, the first stage is microbial attachment. Treating a surface with germ-repelling compounds prevents microbes from adhering to that surface, thus inhibiting microbial colonization on the surface. Numerous mechanisms are involved in microorganism attachment to biomaterial surfaces. All microbial species share some of these mechanisms, whereas others are unique to particular species or strains. A mechanism common to all microorganisms is passive microbial adsorption on biomaterials’ solid surfaces due to physicochemical surface interactions. Species- and strain-specific attachments are active, caused by bacteria, and referred to as bacterial attachments [9,10].

With advancements in its properties (e.g., performance and being a multifunctional material with bioactive properties), antimicrobial coatings are becoming a key component in preventing bacterial infection by stopping bacterial colonization on the surface of medical devices [11] A biomaterial is covered with a layer that incorporates an antimicrobial substance that inhibits the growth and colonization of bacteria, molds, fungi, parasites, germs, and other microbes without affecting the bulk properties of materials in health applications [12]. In addition to protecting biomaterials against microbes, antimicrobial surfaces on biomaterials (e.g., medical devices, surfaces, and implants) reduce the need for disinfectants, are cost-effective, and increase the value and lifespan of biomaterial surfaces [13].

An antimicrobial surface can be fabricated using two methods: (1) modifying the physical properties of the material by altering its material or surface roughness and (2) changing the chemical properties of the material by grafting polymers onto superhydrophobic surfaces and nanomaterials, as well as coating. Antimicrobial coating materials include metal and metal nanoparticles, metal alloys, graphene and its compounds, hydrophobic graphene, dendrimers, and polymer brushes [13]. Based on the physical and chemical interactions between a biomaterial surface and microorganisms, which are responsible for the antimicrobial nature of the surface, antimicrobial surfaces can be grouped into the following types: patterned surfaces; functionalized surfaces; superwettable (superhydrophobic and superhydrophilic) surfaces; and smart surfaces [14].

**Figure 1:**
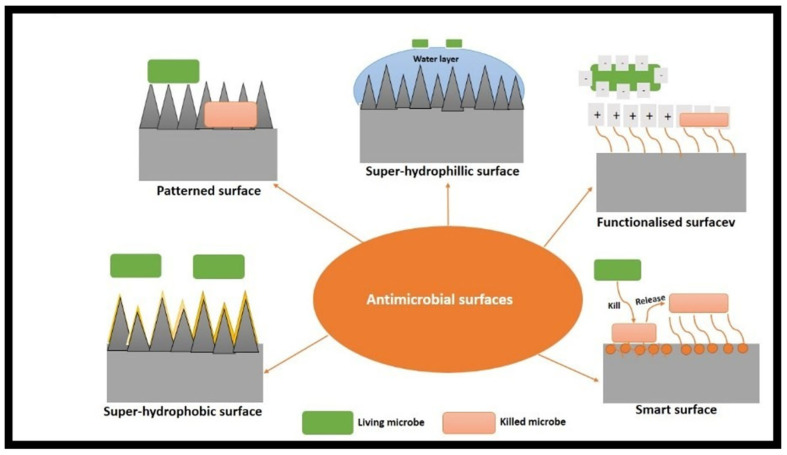
Schematic diagram of different antimicrobial surfaces [11].

Physically modifying materials are used to prepare patterned surfaces. The biocidal mechanism is caused by steric hindrance, which prevents microbes from coming into contact with the surface, or the penetration of the material’s surface features, which results in the physical disruption of the cell and eventual death of microbes [15,16]. Nature has numerous microstructured and nanostructured surfaces that prevent microbe attachment, such as lotus flower surfaces, taro leaves, and shark skin. Some surfaces, such as gecko skin, cicadas, and dragonfly wings, have microbicidal activity [15,17]. Chemical modification is performed to kill microbes or stop their growth when preparing functionalized surfaces. The microbicidal activity of a functionalized surface is caused by the contact-killing of microbes by chemical groups on the material’s surface, by heat generated by the functionalized surface, and by the disruption of microbe activity due to the reactive species caused by exposure to external stimuli [17,18]. A superwettable surface requires a combination of physical and chemical modifications. Material wettability can be enhanced to develop antiadhesive surfaces that prevent biofilm formation by restricting contact between microbes and surfaces. Superhydrophobic and superhydrophilic surfaces can be developed by combining roughness modification and surface chemical treatment. However, superwettable surfaces can only prevent microbe adhesion to surfaces, so they are considered a separate category [19]. Finally, for innovative surfaces, antimicrobial activity is induced by multiple action modes and the switchable property of simultaneously terminating and inhibiting surface microbe growth. This is achieved by incorporating antimicrobial agents into the surface to kill microbes, which are then released using stimuli-responsive polymers [14] (Figure 1).

Antibacterial coatings are designed using the following strategies: release of antimicrobial agents, contact killing, and antiadhesion and bacteria repelling are described in detail in Table 1 [11].

#### 2.1.1. Release-Based Coatings

Release-based coatings are used in a broad range of sectors, including automotive, healthcare, electronics, and packaging. These coatings are ideal for applications such as labels, tapes, and medical devices because of their unique properties, such as excellent release, low surface energy, and high-temperature resistance [21]. The following are some of the current advancements and challenges in the field of release-based coatings:

Coatings that are water-based and solvent-free silicone-based: To comply with demanding environmental laws, manufacturers are focusing on producing ecologically friendly water-based and solvent-free silicone release coatings [21].

Antibacterial coatings: Antibacterial coatings based on release are gaining interest due to their potential in healthcare applications. These coatings can be used to kill bacteria, repel them, or release antimicrobial chemicals [16].

Antimicrobial coatings for healthcare usages: Antimicrobial coatings, like titanium, can be applied to a wide range of surfaces to inhibit bacterial growth while improving the hygiene of healthcare facilities [22]. However, because of the requirement for effective and safe materials, identifying suitable coatings for hospitals can be a major challenge [22].

Releasing and repelling coatings: These coatings can be used for contact-killing and repelling applications, in addition to antimicrobial agent release and repelling [22]. More research needs to be conducted to enhance the performance and safety of these coatings in healthcare settings.

Sustainable options: The research and development of replenishable coatings, such as photocatalytic and N-halamine coatings, is a key to ecologically benign and sustainable solutions [22]. These coatings hold the potential to improve performance while also decreasing the environmental impact.

In a nutshell, release-based coatings have numerous uses in a wide range of sectors, and recent advances include water-based and solvent-free silicone release coatings, antibacterial coatings, and antimicrobial coatings for healthcare applications. The challenges include ensuring the efficiency of these coatings, reducing the risk of bacterial resistance, and adopting the right material for specific applications [16,21,22]

#### 2.1.2. Contact-Killing Coatings

Surfaces that kill microbes on contact are known as contact-killing coatings. They offer a wide range of possible applications, including healthcare, food processing, and dental restorative materials [22,23] Recent advances in this field include the production of hydrogel-based antimicrobial coatings that contain antimicrobial polymers or peptides [24]. Another recent study showed that gram-positive and gram-negative bacteria could be successfully eliminated by hyperbranched antimicrobial coatings adsorbed on polydimethylsiloxane (PDMS) [25]. However, there are still challenges to overcome, such as the difficulty of obtaining long-term antimicrobial surface protection, especially for frequently handled surfaces [23]. Furthermore, additional research is required to prove the clinical application of contact-killing coating in a variety of settings [22].

#### 2.1.3. Anti-Adhesive Coatings

Antiadhesive coatings rely on specific molecules or compounds to prevent microbes, proteins, or other substances from adhering to surfaces [26]. These coatings were developed to be safe and not release any harmful substances when used. Hydrogels, cell adhesive motifs, and self-lubricating liquid layers are some of the molecules and chemical compounds used in antiadhesive coatings [27]. Current challenges and limitations of these coatings include the inability of these coatings to inhibit the growth of pathogenic bacteria, an absence of long-term stability in complex biological environments, and a potential negative impact on the adhesion and development of normal human cells on their surface, which can impair tissue integration [28]. Furthermore, there is frequently a mismatch between the surface chemistry and the intended usage, and certain coatings may require surface pretreatment steps before application [29].

### 2.2. Materials with Antimicrobial Properties

There have been many breakthroughs in the development and fabrication of antimicrobial materials in recent years. The production of carbon-based nanomaterials with antimicrobial properties is showing promise because of an exponential increase in the synthesis of carbon-based metal hybrid nanomaterials [30]. In addition, studies on the application of carbon-based nanomaterials to treat diseases related to bacterial biofilms have shown their promise in tackling antimicrobial issues [31]. There have been advancements in the design, structure, and production of dual-function bio-responsive materials as well as in multifunctional antimicrobial surfaces and their applications in different sectors of industries [32]. Furthermore, the application of the magnetron sputtering method for the fabrication of antibacterial metal surfaces has been explored, showing the ongoing research in metal-based antimicrobial materials [33]. Metal-based antimicrobial coatings, particularly those based on copper, silver, and zinc, are gaining attention worldwide for their capacity to stop the transmission of viruses, bacteria, and fungi through high-contact-level human surfaces [34]. These coatings have been applied to a variety of objects, like fabrics, doorknobs, and handrails, because of their effective antibacterial action [35]. A great deal of research has also been conducted to develop biomaterials and biomedical devices that are antimicrobial and antibiofouling, with an emphasis on qualities that are antibacterial, antibiofilm, and antibiofouling against microorganisms and other biomacromolecules [36]. Moreover, research on multifunctional antimicrobial surfaces is continuing, and the development of antibacterial metal surfaces and the magnetron-sputtering method have been explored as possible means of advancing antimicrobial materials.

The possibility of antimicrobial resistance, which can happen when microbes become immune to antimicrobial agents over time, is one of the challenges in the development of antimicrobial materials [35]. The possibility of antimicrobial resistance, which can happen when microbes develop immunity to antimicrobial agents over time, is one of the challenges in the development of antimicrobial materials [35]. Furthermore, while producing safe and effective antimicrobial compounds, the possible toxicity of some antimicrobial materials, especially those that include heavy metals, must be considered [35].

The use of metal-based nanoparticles (NPs) for antimicrobial purposes has been the focus of recent research. These NPs may consist of pure metals (such as gold, silver, or iron) or compounds like oxides [37]. However, challenges in this field include understanding the toxicity mechanism of metal-based nanoparticles (NPs), mainly concerned with forming reactive oxygen species and disrupting membrane function [37]. Further research to advance the application of antimicrobial materials to clinical use needs more study because of the limited assessment of metalloantibiotics with in vivo animal models [38]. A lack of advancements in bringing metalloantibiotics to the clinic, despite the historical application of metals such as copper for antibacterial purposes, has met regulatory and approval obstacles.

Antimicrobial applications have shown promise for carbon-based nanoparticles, such as carbon quantum dots, nanotubes, and two-dimensional materials like graphene. Nevertheless, challenges in this field include not fully knowing the exact mechanisms of the bactericidal action of carbon-based NPs, demonstrating a need for further research in this area [37]. Although carbon-based NPs have shown promise in preventing the formation of biofilms, further research is needed to determine the precise mechanism at play [37]. Understanding toxicity mechanisms, having limited clinical evaluation, and gaining regulatory approval are the main challenges researchers face while producing metal-based antimicrobial compounds. Understanding mechanisms of action and comprehending the effectiveness of carbon-based antibacterial compounds in preventing biofilms are challenges. It will be essential to address these problems if these antimicrobial materials are to be developed in various applications [37].

Different materials possess intrinsic antimicrobial properties for bioengineering applications. These include ceramics (e.g., zinc oxide [ZnO], manganese peroxide, and titanium dioxide [TiO_2_]), polymeric materials (e.g., chitosan [CS]) and their related compounds, and metals (e.g., silver, zinc, and copper). Table 2 summarizes the substances typically employed as antibacterial agents and their modes of action.

## 3. Antimicrobial Mechanism and Properties of Metal and Metal Nanoparticles

Metals, such as silver, gold, and copper, have biocidal properties and are recognized for their antimicrobial capability. They are also used for in vitro and in vivo applications [58]. Here, the general mechanism of metal and metal nanoparticles, which are the most commonly used antimicrobial materials, their antimicrobial activities, and related mechanisms are briefly discussed.

### 3.1. Structure of the Bacterial Cell Wall

The structure of the bacterial cell wall should be understood for a better comprehension of the mechanisms of metal ions and nanoparticles. The bacterial cell wall has a multilayered mesh-like structure consisting of proteins, lipids, and carbohydrates. Based on gram staining and cell wall differences, bacteria can be classified as gram-positive or gram-negative bacteria [59,60].

In gram-positive bacteria, the cell wall, which is a thick peptidoglycan layer (20–80 nm), consists of repeating units of N-acetyl glucosamine-N-acetyl-muramic acid cross-linked by pent peptide side chains, forming a robust structure. Teichoic acid is attached to the peptidoglycan layer [60]. Gram-negative bacteria have a more complex cell wall structure with a thin peptidoglycan layer (7–8 nm) between the cell wall and the outer membrane. Moreover, gram-negative bacteria have negatively charged lipopolysaccharides in their outer membranes. Despite containing channels that allow specific molecules to enter, such as porin channels, the outer membrane blocks the entry of macromolecules. The pathogenicity of gram-negative bacteria is significantly influenced by the lipopolysaccharide endotoxin [61].

Gram-negative bacteria are less vulnerable to metal ions and their nanoparticles than gram-positive bacteria because their outer membranes are less permeable. Furthermore, gram-positive bacteria do not possess the cell envelope. However, gram-positive bacteria such as *Staphylococcus aureus* are less sensitive to copper and silver compared with gram-negative bacteria. However, it is the composition and thickness of individual cells, rather than the Gram type and cell wall structure, which dictate bacterial sensitivity to metals important for antibacterial activity [59].

### 3.2. General Mechanism of Antibacterial Activity of Metal and Metal Nanoparticles

Various physical and chemical characteristics of metal ions determine their mechanism of toxicity to microbial cells. Metal ions can interact with multiple targets in a bacterial cell, including enzymes, molecules, and membranes. They are observed in various chemical compounds, depending on the local environment’s temperature, pH, ionic strength, and reduction potential. For example, the reducing environment of the cytoplasm of a gram-negative bacterium is more vital than that of the periplasm of the same bacterium, which consequently affects a metal’s oxidation state and speciation and influences its bioavailability and reactivity; this is a significant physicochemical property of metal toxicity [45].

**Figure 2:**
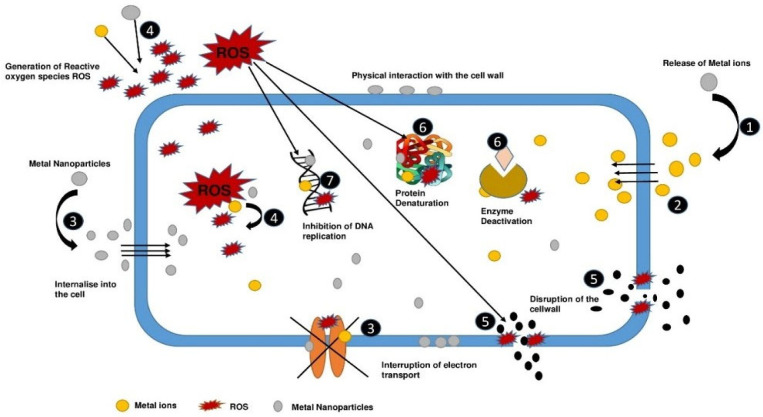
Schematic diagram of various antimicrobial mechanisms of metal ions and nanoparticles.

Inorganic particles with different shapes and sizes ranging from 0.001 to 0.1 µm are called metallic nanoparticles. The antibacterial property of these metal nanoparticles differs depending on various aspects, such as size, charge, zeta potential, surface structure, and morphology [62]. For instance, the smaller the metal nanoparticle, the greater its antibacterial activity and vice versa. This phenomenon is because of its large surface-to-volume ratio, which increases its capacity to produce ROS and ultimately damages the biomolecules of the bacteria, leading to cell death. If two metal nanoparticles have the same surface-to-volume ratio, then the shape of the metal nanoparticle plays the determining role in antimicrobial activity. Metallic nanoparticles possess unique properties because their high surface area to volume ratio and shape influence their properties [59]. The shape of metallic nanomaterials significantly impacts many aspects of the materials, such as antimicrobial, electromagnetic, optical, and catalytic capabilities. Rods and nanotubes are more efficient than other shapes because of metal oxidation and the position of their planes [59] (Figure 2).

The broad antibacterial mechanisms of metals and metal nanoparticles can be categorized as follows: (1) release of metal ions from metal nanoparticles; (2) direct interaction of metal ions with the cell wall; (3) interaction of metal nanoparticles and the cell wall via electrostatic attraction, which compromises membrane function and nutrient assimilation; (4) ROS generation inside and outside cells; (5) damage caused by oxidative stress to lipids, proteins, and DNA; and (6) substantial amounts of metal binding to the cell envelope along with elevated ROS levels, which can damage the plasma membrane and lead to the leakage of the cell contents. Metal ions and nanoparticles can directly affect DNA and protein functioning upon metal absorption [5,59], which directly affects cellular metabolism and metal-mediated ROS generation.

### 3.3. Common Metals and Metal Nanoparticles Used as Antimicrobial Agents

#### 3.3.1. Silver

Despite being used to treat illnesses, silver and its associated chemicals were only identified as possessing antibacterial properties in the late 1800s. Silver ions are toxic to bacteria, fungi, viruses, and other microorganisms, but have little to no effect on humans [2,3]. Silver ions are widely used antimicrobial agents for medical applications. They are used in various medical devices because of their potent antimicrobial action [2]. Ag NPs have advantageous properties; for example, they can be synthesized in a way that allows for precise control of their size, shape, and structure, and their biochemical functionality can be tailored for a specific application [59]. Thus, Ag NPs are attracting increasing attention in many scientific fields for various applications [59].

In addition to exerting antibacterial action, Ag NPs exhibit ionic silver characteristics due to the spontaneous release of silver ions from the nanoparticle surface. Ag and Ag NPs also function as antiviral agents by deactivating viruses through interactions with viral surface proteins and envelopes, preventing viral entry into cells, and blocking cellular pathways through interactions with viral genomes and replication factors [5]. Using the HIV-1 strain to explain the viral inactivation mechanism of silver, Lara et al. showed that Ag NPs function as early-stage antivirals that prevent viral multiplication. They also suggested that by binding to or fusing into cells, Ag NPs inhibit viral entry into cells and disrupt viral replication at later stages, albeit through an unconfirmed mechanism [5].

Silver has been utilized as an antibacterial substance for a long time, and the current study has concentrated on its applicability in various industries. Silver has been used in treatments and medicinal items for its bactericidal function, with increased interest due to the increasing prevalence of bacterial resistance to antimicrobials. With a novel technology for making ready-packed medical equipment that sterilizes itself upon opening, silver-based surface coatings are widely employed in medical applications [63]. The antibacterial properties of silver are widely appreciated in implanted devices. Silver nanoparticles can also be used to coat invasive surgical equipment such as medical-grade needles. Vascular catheters, bone implants, and biliary duct brackets are examples of silver-containing medical devices that are directly injected into the human body [63].

Silver has unique qualities, which have been used in medicine, notably in wound treatments for troops during World War I, to inhibit microbiological development [2]. Furthermore, silver ion antimicrobial textiles used in sporting and fashion clothes, beds, accessories, and masks have been created to provide antibacterial protection. These examples show how silver may be used as an antibacterial material in various real-world scenarios, including medical treatments and ordinary items [64].

#### 3.3.2. Zinc

At low concentrations, zinc ions benefit cells in multiple ways. They play a crucial role in regulating cellular proliferation and differentiation and act as cofactors in various metabolic pathways. At high concentrations, they inhibit microbial cell growth [65]. Various research findings have indicated that the antimicrobial activity exhibited by zinc nanoparticles is primarily attributed to the release of zinc ions into the surrounding medium [66,67]. The efficiency of zinc as an antiviral agent against the human rhinovirus was demonstrated by Korant et al. (1974) using 0.1 mM of zinc chloride, which resulted in a 99.9% decrease in the plaques produced by the human rhinovirus [5].

Zinc has broad-spectrum antibacterial effects that inhibit fungus and bacteria development. It is used in paints, textiles, and polymers to suppress the development of germs and fungi on porous surfaces and fabrics [68]. Zinc chloride has been proven to have antibacterial and antibiofilm properties, making it helpful in avoiding infections caused by medical devices [62]. Furthermore, zinc oxide nano/microparticles have been investigated as antibiotics to improve antibacterial activity against pathogenic bacteria and viruses with and without antibiotic resistance [69]. Zinc oxide is a good material for antimicrobial coatings in biomedical applications because of its minimal toxicity, great biocompatibility, durability, and affordability [70]. Furthermore, it has been demonstrated that the deposition of zinc oxide on various polymer fabrics imparts antibacterial qualities, making them appropriate for daily applications such as sportswear and medical textiles [71]. These examples show how zinc may be used as an antibacterial material in a variety of real-world environments, including healthcare, textiles, and biological applications. Zinc oxide is a good material for antimicrobial coatings in biomedical applications because of its minimal toxicity, great biocompatibility, durability, and affordability [71].

#### 3.3.3. Copper

Copper has long been used in medicine as an antibacterial and anti-inflammatory material, making it the most well-known antimicrobial metal to date [72]. According to modern research, the antimicrobial mechanisms of copper are as follows: (1) plasma membrane permeabilization, (2) lipid membrane peroxidation, (3) protein alteration, (4) protein assembly and its activity’s inhibition, and (5) nucleic acid denaturation. The electrostatic forces applied by copper ions on the outer plasma membrane can disrupt the membrane. The interaction of essential metals with proteins or displacement of necessary metals from their binding sites on proteins damages these proteins. The cyclic redox reaction between copper ions produces ROS, ultimately leading to microbial death [5]. Research on the virucidal nature of copper demonstrated that it targets viral genomes, especially the genes responsible for viral infection. Numerous researchers have hypothesized that the ROS mechanism that confers antimicrobial activity can also affect the viral envelope. Viruses lack the repair mechanisms found in bacteria and fungi, making them vulnerable to copper-induced damage [5].

To understand the effect of copper surfaces, various studies of copper and copper alloys used in experiments using a dry and wet inoculation technique to stimulate a clinical setting have been performed to understand the antimicrobial effects on various bacteria, viruses, fungi, and yeasts [73]. The biocidal efficacy depends on the concentration of copper, exposure time, humidity, and temperature. Keevil’s research team in Southampton, UK, conducted experiments with different concentrations of bacteria and exposure times to achieve the most efficient biocidal effect [74]. They found that biocidal efficacy increases with higher copper concentrations. Copper continuously reduced microbial contamination, with a 99.9% reduction within 2 h of exposure. Initial studies used a wet inoculum at high relative humidity and high temperatures [2]. However, different laboratory techniques were used, such as a dry inoculum or wet inoculum, reflecting touch contamination. Bacterial killing occurred more rapidly under dry conditions than under moist ones [73]. Using a wet inoculum, bacteria like Enterococcus faecalis or Enterococcus faecium were killed in 1 h, compared to 10 min when presenting the inoculum to metal coupons in lower volumes. The effects of temperature and relative humidity were also explored [73].

Studies suggest that soft surfaces in healthcare environments, such as white coats and bed linen, maybe a potential hazard for cross-contamination due to the horizontal transmission of pathogens through hard surfaces [73]. To address this issue, copper has been introduced into textiles and liquids, with recent developments in nanotechnology allowing the impregnation of textiles, latex, and other polymer products with copper oxide [2]. Metal nanoparticles have enhanced antimicrobial ability due to their high surface area compared to their volume, allowing cell membranes to be more rapidly penetrated [2]. Laboratory studies have shown the broad-spectrum antimicrobial properties of copper-impregnated fibers, which can be used in products such as socks, face masks, filters, bed linen, and scrubs [73]. A research team in Israel reported on copper-impregnated products and their effectiveness against bacteria, fungi, and viruses, showing a >2-log reduction within 2 h of exposure [75].

Copper has been extensively researched for its antibacterial qualities and used in various real-world applications. Copper has been examined in healthcare facilities for use on contact surfaces such as door handles, bathroom fixtures, and bed rails to prevent hospital-acquired infections [76]. Solid copper surfaces have been proven by in vitro studies to kill 99.9% of microbes within two hours of contact, with a magnitude of 7 to 8 logarithms per hour, and no bacteria are recovered after prolonged incubation periods [77].

#### 3.3.4. Other Metals

Other than silver, zinc, and copper, some of the other commonly known metals and nanoparticles known for their antimicrobial activity are gold, platinum, magnesium, and aluminum. The formation of reactive oxygen species has reported the antimicrobial activity of the gold nanoparticles, which may lead to oxidative damage to the bacterial cell membrane and DNA [78]. Platinum nanoparticles have been reported to disrupt bacterial redox balance, leading to bacterial homeostasis disruption and, ultimately, bacterial death [79]. The breakdown of bacterial cell membranes and inhibition of bacterial cell wall formation have demonstrated the antimicrobial activity of magnesium nanoparticles [80]. Aluminum nanoparticles have been reported to have antimicrobial action by generating reactive oxygen species, which can induce oxidative damage to the bacterial cell membrane and DNA [81]. In a recent study by Pitkel et al. [82], it was found that gold nanoparticles with different shapes, like spheres, rods, stars, and nanocapsules, have been demonstrated to exhibit potent antimicrobial activity against a wide range of microbial pathogens, including fungi such as Candida albicans, gram-negative bacteria, and gram-positive bacteria [82]. In another study by Vukoja. et al. [81] found that colloidal platinum nanoparticles were shown to have significant antibacterial effects against standard laboratory as well as resistant strains of *Escherichia coli* and *Klebsiella pneumonia*, demonstrating their potential antimicrobial efficacy [83]. In recent research findings, it was revealed that antimicrobial magnesium nanoparticles show excellent antimicrobial activity against a wide range of pathogenic bacteria, whereas findings on the antimicrobial activity of aluminum nanoparticles are limited. A comprehensive review of metal-based nanoparticles has focused on the non-specific bacterial toxicity mechanisms of aluminum nanoparticles, making bacterial resistance formation difficult and broadening their spectrum of antibacterial action [79,83].

## 4. Antimicrobial Mechanisms and Properties of Polymers

Antimicrobial polymers can slow down or stop microbial growth, combat antibiotic-resistant bacteria, and easily coat or sterilize biomaterial surfaces, especially medical equipment [84]. Biopolymers and synthetic polymers have antimicrobial polymeric systems. Except for CS, polymers do not have intrinsic antibacterial properties. Polymeric antibacterial materials are prepared by modifying polymer matrices using antimicrobial or antibacterial agents through different methods [85]. Various surface modification techniques are used to produce surface-assisted antimicrobial properties. Chemical surface modifications include derivatization, surface functionalization, and polymerization, whereas physicomechanical modifications include mechanical and surface structuring [86]. Antimicrobial activity can be induced by grafting functionalized polymers or implementing physicochemical adsorption. Antimicrobial agents containing different antimicrobial polymers (on the substrate surface), enzymes, and polymers are immobilized to provide antimicrobial effects. Most synthetic and natural antimicrobial polymers are well-known [3]. CS is a cationic and polycationic polymer, which exhibits antibacterial activity by interrupting the net negative charge of the bacterial cell membrane, causing the cell to lyse and eventually die. Table 3 summarizes the different classifications of polymer-based antimicrobial mechanisms. These mechanisms are mostly related to polymeric substances that are drug-loaded, created in hydrogels, or bonded to surfaces [61].

### CS

**Figure 3:**
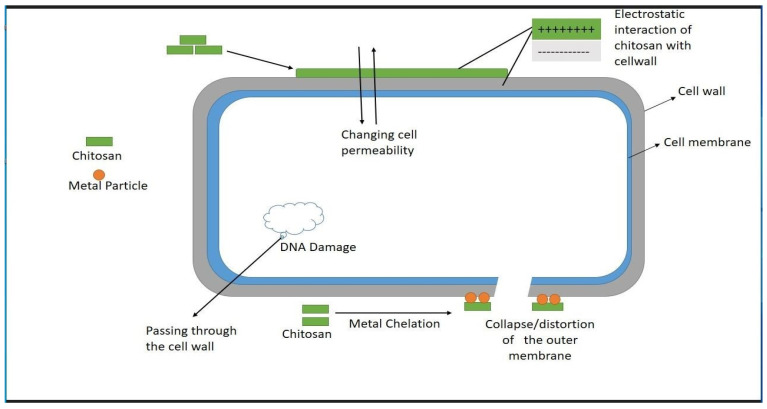
General schematic diagram of antimicrobial mechanism of CS [86].

Because of its natural antimicrobial characteristics, extensive sources, and high yield, CS is a preferable antimicrobial material. Its antimicrobial properties are due to its cationic nature. Although the specific mechanism of its antimicrobial action remains unidentified, various hypotheses have been proposed to explain its antimicrobial nature. First, bacterial biofilm splitting, which may be due to the interaction between positively charged CS molecules and negatively charged microbial cell walls, leads to the leakage of the resultant proteins and other cell elements, ultimately resulting in microbial death [46]. Second, chitosan acts as a chelating agent by selectively binding metal proteins and inhibits the formation of toxins and development in microorganisms. Third, chitosan combines with and triggers the cell membrane component, leading to the breakdown of the cell membrane and, ultimately cell death. Fourth, more chitosan and DNA are introduced into the nuclei of microorganisms, and this chitosan in the nuclei inhibits the mRNA and protein activity [13] (Figure 3).

Chitosan, a naturally occurring biopolymer generated from chitin, has undergone substantial research for its antibacterial characteristics and has found practical uses in a variety of industries. Here are some real-world instances of chitosan’s use as an antibacterial material: Biomedical and food packaging applications have benefited from the development of chitosan-based films. These coatings have been found to limit the growth of spoilage-causing microbes, hence prolonging food product shelf life [86]. Chitosan has antibacterial qualities and film-forming abilities, making it an appealing biopolymer for antimicrobial food packaging and preservation applications [94] Chitosan has been utilized to make textiles, films, fibers, membranes, and hydrogels for various purposes, including antimicrobial applications in the textile and fashion industries [46]. Due to its antibacterial characteristics, chitosan has been studied for possible biological and pharmaceutical uses. The focus of research has been on developing chitosan-based systems for diverse antibacterial applications [46,47]. These examples show the various practical applications of chitosan as an antibacterial material in real-world contexts, such as food packaging, textiles, and biomedical applications.

## 5. Antimicrobial Mechanism and Properties of Ceramics

**Figure 4:**
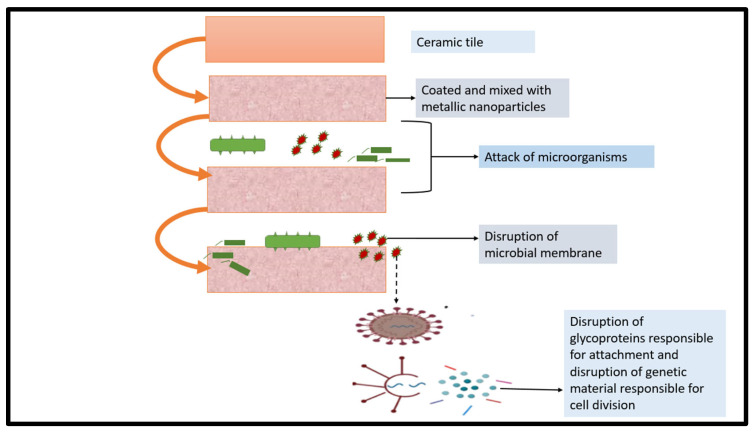
General schematic diagram of antimicrobial mechanism of ceramics [44].

Antimicrobial ceramics are used in various biomedical devices, such as catheters, vascular grafts, and orthopedic implants. A typical example of antimicrobial ceramics is hydroxyapatite, which has been widely investigated to replace hard tissues because of its good biocompatibility and good integration into tissues and bones [3]. Combined with silver, ZnO, and CuO, hydroxyapatite has been reported to improve antimicrobial activity [3] (Figure 4).

### 5.1. ZnO

ZnO is an efficient antimicrobial agent used by many pharmaceutical and cosmetics companies [43,44]. It acts as an antimicrobial agent using different chemical species through several mechanisms as follows: (1) ROS production caused due to the semiconductor properties of ZnO; (2) direct contact of ZnO particles with the microbial cell wall, which destabilizes the microbial membrane; and (3) zinc ions, which have intrinsic antimicrobial properties, released by ZnO in aqueous solutions [41]. Despite various concerns surrounding their antimicrobial efficiency, the antibacterial property of zinc oxide nanoparticles has attracted worldwide attention, which may be linked mainly to the use of nanotechnology, which produces particles in the nanometer range.

The specific toxicity mechanism of ZnO nanoparticles remains unknown, and many issues about their antimicrobial effectiveness warrant in-depth explanations. According to several mechanisms described in the literature, microbial cell integrity is destroyed by the direct interaction between ZnO nanoparticles and the cell wall as well as by the release of zinc ions, antimicrobial ions, and ROS [95]. However, the toxicity mechanism differs between different solutions. The dissolved zinc species may vary depending on the properties of the medium and the physicochemical properties of ZnO nanoparticles. Bioengineering applications use ZnO nanoparticles. For instance, Varaprasad et al. used precipitation to prepare ZnO nanoparticles, which were then impregnated over cellulose fibers using a sodium alginate (SA) matrix. The antibacterial potency of the ZnO nanoparticle–SA cellulose fibers against *Escherichia coli* was tested. Standard antimicrobial test results showed that the specimen with a microbial zone inhibition of >1 mm was a good antimicrobial agent [96].

Zinc oxide (ZnO) has been extensively researched for its antibacterial characteristics and has practical uses in a variety of real-world scenarios. Here are some instances of how zinc oxide has been used as an antibacterial material: Antimicrobial coatings for medical equipment and biomedical applications have been developed using zinc oxide. Antimicrobial qualities have been demonstrated in these coatings, making them appropriate for usage in healthcare institutions to reduce the spread of infectious microorganisms [69]. Zinc oxide nanoparticles have been used as an antibacterial agent in food packaging to prevent foodborne infections. The antibacterial activity of nanosized ZnO has been investigated for potential use in food packaging to improve food safety and shelf life [95]. Zinc oxide has been added to textiles, polymers, and other daily items to limit the growth of germs and fungus on vulnerable surfaces and fabrics. This treatment improves the efficacy of standard cleaning procedures and supplements the use of disinfectants [68]. These examples show how zinc oxide may be used as an antimicrobial material in various real-world applications, including healthcare, food packaging, and daily objects.

### 5.2. TiO_2_

Titanium oxide (TiO_2_) has excellent antimicrobial activity and is effective against gram-positive and harmful bacteria, viral species, parasites, and spores of *Bacillus*, a resistant organism [4]. Light absorption, electron generation, and ROS causing organic material oxidation are the mechanisms of pathogenic inactivation related to TiO_2_ [5]. TiO_2_ nanoparticles are the most studied antimicrobial agents because of their unique qualities such as bactericidal photocatalytic activity, safety, and self-cleaning capability [4]. The antimicrobial mechanism of titanium oxide is related to ROS, which affects the microbial cell through various mechanisms that ultimately lead to cell death. Chen et al. used CS/Sr–TiO_2_ polymeric dressings with antibacterial solid activity to treat wounds. The wound-healing abilities of CS/Sr–TiO_2_ were evaluated on rabbit joint wounds; the material was found to be effective, exhibiting an impressive wound-healing rate of 93% after 12 days [4].

Antimicrobial coatings for medical equipment and biological applications have been developed using TiO_2_ nanoparticles. Antimicrobial qualities have been demonstrated in these coatings, making them appropriate for usage in healthcare institutions to reduce the spread of infectious microorganisms [13,97]. TiO_2_ nanoparticles have been used as an antibacterial agent in food packaging to combat foodborne infections. The antibacterial activity of nanosized TiO_2_ has been investigated for possible use in food packaging to improve food safety and shelf life. TiO_2_ has been integrated into textiles, polymers, and other daily items to limit the growth of germs and fungi on vulnerable surfaces and fabrics. This treatment improves the efficacy of normal cleaning procedures and supplements the use of disinfectants [98]. A recent study has concentrated on the green manufacturing of TiO_2_ nanoparticles utilizing plant extracts such as Luffa acutangula leaf extract and rosemary and ginger extracts to improve their antibacterial activity [97,99]. These examples show how TiO_2_ may be used as an antimicrobial material in a variety of real-world applications, including healthcare, food packaging, and daily objects.

## 6. Antimicrobial Activity of Carbon-Based Materials

A metal-based material induces cytotoxicity in mammalian cells, necessitating research on more biocompatible materials, such as carbon-based ones (e.g., graphite, graphene, diamond, and CNTs) [100]. Carbon nanostructures (CNs) are highly efficient in stopping or inhibiting microbial growth because they have powerful bactericidal properties. The antimicrobial mechanism of CNs depends on their various intrinsic properties, including their surface composition and modification, the type and nature of the target microbe, the environment of the cell–CN interaction, and environmental characteristics [101].

**Figure 5:**
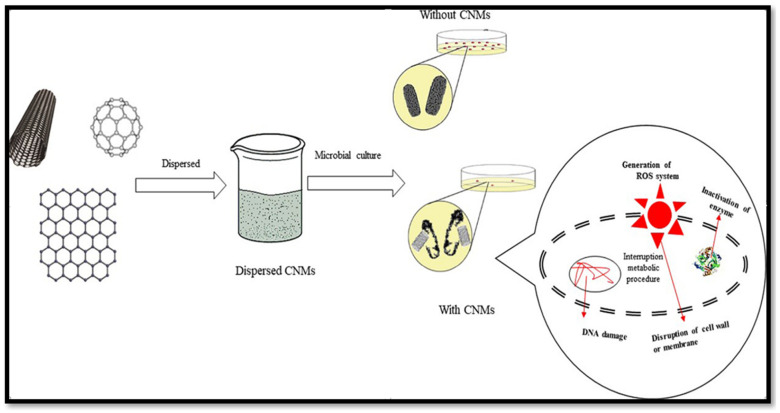
General schematic diagram of antimicrobial mechanism of carbon-based materials [105].

Physical and chemical mechanisms are responsible for the bactericidal action of CNs. As for the physical mechanism, CNs damage the cell wall structure of microbes. Carbon nanomaterials, such as graphene sheets, can biologically isolate microbial cells from their microenvironment, resulting in cell death [102]. Chemical interactions between CN surfaces and microorganisms lead to the formation of harmful chemicals (e.g., ROS) that place cells under oxidative stress. Such an interaction between CNs and microorganisms may result in electron transfer. In this process, electrons are gradually drained from the outer surface of the bacteria, causing non-ROS-induced oxidative stress and then microbial death [103,104] (Figure 5).

### 6.1. CNT

Both SWCNTs and MWCNTs strongly inhibit various microbes, even after brief exposure. Therefore, CNTs are effective antimicrobial agents, which can be used for biomedicine applications [106,107,108]. The microbicidal effect of SWCNTs is better than that of MWCNTs. However, the antimicrobial mechanism of CNTs is not fully understood [109]. The diameters, lengths, residual catalysts, electronic structures, functional groups, coatings, and surface chemistry of CNTs are some of the variables that determine their antibacterial properties [110]. The following antibacterial mechanisms of CNTs have been proposed: (1) electrostatic forces between the outer surface of microbes and CNTs, causing membrane oxidation and thus membrane disruption; (2) inhibition of the biological molecules of bacteria or DNA destruction caused by ROS; and (3) impurities introduced into the structure during CNT production, which can contribute to antibacterial activity [111].

Several investigations have demonstrated that carbon nanotubes have potential antibacterial capabilities. For example, functionalized multiwall carbon nanotubes (FMWNTs) have been studied for their antibacterial action against harmful microbes such as *E. coli* and *S. aureus* 1. Carbon nanotubes have also been shown to have high antibacterial action and the ability to puncture bacterial cell walls [111]. Furthermore, a study found that carbon nanotube-containing chemical compounds have an antibacterial impact on drug-resistant Acinetobacter baumannii isolates [112]. Another recent discovery involves the creation of a nanoantibiotic based on the coupling of multi-walled carbon nanotubes with levofloxacin, which has shown strong antibacterial activity in in vitro and in vivo investigations [105]. Furthermore, carbon nanotubes have been investigated as antimicrobial agents for water disinfection and disease control due to their potent antibacterial capabilities [100,113]. Finally, research found that highly pure single-walled carbon nanotubes have considerable antibacterial activity. These findings emphasize carbon nanotubes’ promise as antibacterial materials and recent advances in this field [111,114].

### 6.2. Graphene

Numerous compounds related to graphene have been studied. These include graphite, GO, reduced GO, GO nanosheets, multilayer graphene, and virgin graphene [115]. The exact antimicrobial mechanism of graphene is difficult to predict because of the intrinsic properties of graphene materials and their related compounds [116]. Accordingly, various scenarios have been examined to understand the antimicrobial mechanism of graphene-related nanomaterials. The proposed antimicrobial mechanisms of graphene and its related materials are as follows: (1) severe insertion and cutting of cell membranes; (2) destructive extraction of phospholipids from lipid membranes [117]; (3) oxidative stress caused by ROS, which extensively damages lipids and proteins (cellular components) [118]; (4) non-ROS-induced oxidative stress caused by oxidation and disruption of critical biological structures due to graphene interference with unique bacterial processes [119]; and (5) trapping and isolation of microbial cells from their environment. The physical size, length, and surface area of graphene nanosheets affect their antibacterial activity [119].

Graphene and graphene-based materials have been shown in several investigations to have substantial antibacterial capabilities. These compounds exhibit antibacterial action against both gram-positive and gram-negative bacteria [120]. Graphene’s usefulness as an antibacterial agent has been demonstrated in various applications, including producing hybrid composites and as a catalyst in catalysis [121]. Graphene-based nanomaterials have also been recognized as potentially valuable ingredients for medicinal applications, notably as antibacterial agents [122]. Graphene oxide (GO), a graphene derivative, has also shown antibacterial action against multidrug-resistant superbugs isolated from sick people [123]. These findings highlight the promise of graphene and graphene-based materials as antibacterial agents, as well as recent advances in this field.

### 6.3. DLC

Diamond-like structures are extensively studied because of their role as an excellent protective coating in biomedical applications. Sp2 and sp3 hybridization is responsible for bacterial adhesion to the DLC. The antibacterial performance of the DLC can be increased by decreasing the sp3/sp2 ratio [115]. Because of its better interaction with human cells, and strong wear and corrosion resistance, DLC with a high sp3 bond fraction is preferred for biomaterial coating [117]. Many variables, including smoothness, the dispersive component of the surface energy, and hydrophobicity, affect the antibacterial action of the DLC [116]. The proposed antimicrobial mechanisms of DLC structures are as follows: (1) intense impairment of microbe membranes and release of microbial intercellular metabolites, leading to direct physical damage to microbes [119]; (2) antibiofouling antibacterial effect of DLC films based on their surface profiles; (3) the unique properties of DLC films, which depend on the circumstances during DLC structure production; and (4) the sp3/sp2 ratio, which is crucial for the biological functions of DLC structures [119].

In numerous applications, diamond-like carbon (DLC) has been researched for its anti-bacterial characteristics. For example, DLC surfaces containing silver nanoparticles have been studied for their biological characteristics and antibacterial effectiveness against harmful microbes [124]. Furthermore, diamond-like carbon–metal composite films have been demonstrated to have increased hardness, corrosion resistance, and antibacterial properties [125]. In addition, DLC films doped with zirconium oxide nanoparticles have been produced as nanocomposites for improved antibacterial characteristics for medical applications [126]. Another recent discovery is the creation of an antibacterial bandage made of diamond-like carbon and silver nanoparticles (DLC: Ag)-coated synthetic fabrics, which have shown potential antimicrobial capabilities [57] Furthermore, fluorinated diamond-like carbon (F-DLC) coating has been demonstrated to be biologically safe, have excellent antibacterial characteristics, and hold promise in preventing postoperative infections [127].

## 7. Application of 3D-Printed Antimicrobial Materials

**Figure 6:**
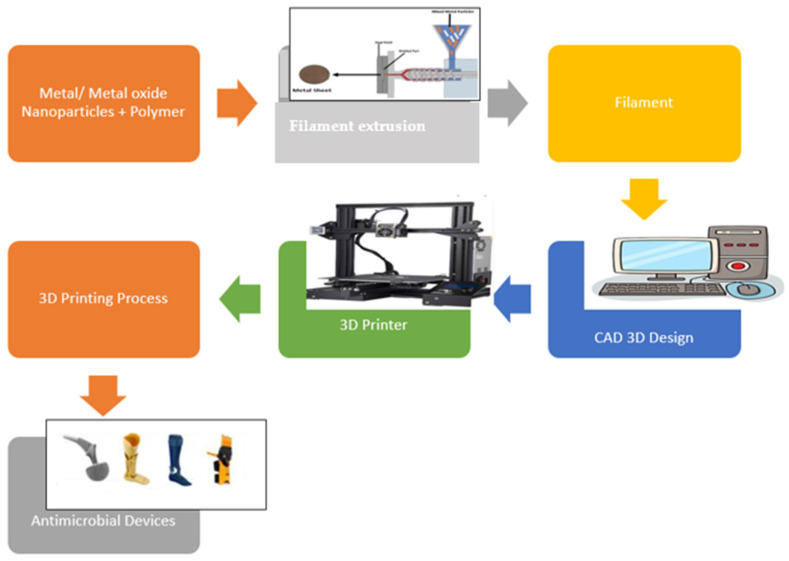
General schematic diagram of 3D-printed antimicrobial materials [129].

Over the past three decades, 3D printing has become a game-changing technology for rapid production and prototyping. Different AM techniques, such as binder jetting, material extrusion, powder bed fusion, and vat polymerization, have been applied in many industries, including tissue engineering and biomedical research [128] (Figure 6).

With the capacity to combine different materials and compositions, 3D printing enables controlled deposition and patterning of polymeric or composite biomaterials. The construction of 3D constructs for tissue engineering is being performed increasingly via 3D printing and bioprinting. 3D-printed biological materials are called biomaterial inks. Biomaterials can be deposited on precise 3D scaffolds using 3D printing technology to create living tissue-engineered constructions. This technology can also be used to develop precise structures with various mechanical and biological qualities. Bioprinting is possible using 3D printers with simple-to-complex configurations and is becoming increasingly affordable, reliable, and user-friendly [130]. However, only a few biomaterial inks and bioinks are widely available, have acceptable printability, and function well against microorganisms when used in bioprinting and 3D printing [6].

The limitations of the materials used in the medical industry, primarily for bone applications, have led to advancements during the past 20 years, particularly in developing synthetic bone material substitutes. 3D printing for bone, dental, and tissue restoration has significantly expanded because of the simplicity of testing, prototyping, and producing personalized, tailored outputs. 3D printing is used to build osteoconductive, biocompatible materials with the porous structure needed to support drug administration and is perfectly compatible with the growth of the surrounding capillaries. Alternative bone materials or scaffolds should be designed to provide the necessary mechanical support, become resorbed into new bones over time, and promote the development of the surrounding tissues [131,132].

Copper compounds have strong antipathogenic capabilities and are widely used in medicine. Copper particles are superior alternatives to other substances (e.g., silver), which sometimes cause skin irritation [133]. They can help design low-cost medical devices with antibacterial solid capabilities. According to several studies on 3D-printed medical prostheses for the upper limbs, including the arms, shoulders, and fingers, 3D printing can offer affordable, adaptable solutions that may include necessary antibacterial surfaces [132,133].

Synthetic polymers are frequently used in bone tissue engineering because of their adaptable physical characteristics and biocompatibility. However, most of these polymers have subpar antibacterial capabilities by nature. Antimicrobial polymers should be developed because infections at implantation sites lead to the failure or delay of the bone healing process [132]. Silver has been used frequently as an antibacterial ingredient to create antibacterial materials in recent years.

Radhakrishnan et al. used a polycaprolactone (PCL) solution to synthesize Ag NPs via in situ reduction, followed by the extrusion of PCL/Ag Np filaments [128]. They used PCL/Ag Np filaments to create customized 3D structures through 3D printing. Scanning electron microscopy revealed interconnected porous networks on 3D-printed scaffolds, whereas X-ray photoelectron spectroscopy demonstrated silver ion reduction. The production of Ag NPs throughout the PCL matrix was verified through energy-dispersive X-ray spectroscopy analysis and transmission electron microscopy. An examination of the in vitro enzymatic degradation of the PCL/Ag NP scaffolds showed an 80% breakdown in 20 days. The cytocompatibility of the scaffolds was determined using hFOB cells, and *E. coli* was used to demonstrate the antibacterial activity of the material. Overall, the study demonstrated that the scaffolds produced may be used to create antimicrobial scaffolds for bone tissue creation [132].

In addition to encapsulating silver into polymers, adding silver ions to ceramics is a well-known method for creating antibacterial ink. This was performed by Wang et al. [134], who incorporated silver into 3D printing inks to create antibacterial constructs for bone tissue engineering. In this study, silver ion-infused zinc silicate zeolite scaffolds (Ag-3DPZS) were effectively built using 3D printing. According to compression strength tests, the Ag-3DPZS printed using attapulgite as an inorganic binder showed outstanding mechanical qualities and could be used as a potential biomaterial for cancellous bone. In vitro investigations revealed that the outstanding biomineralization capacity and bioactivity of Ag-3DPZS can stimulate apatite production. The antibacterial effectiveness of Ag-3DPZS against *S. aureus* and *E. coli* indicated its extraordinary ability to inhibit bacterial growth when applied. All findings strongly implied that the silver-functionalized zinc silicate zeolite scaffolds created via 3D printing had the qualities of implant materials and represented a new option for bone implantation [134].

In Zhang et al.’s study [135], the initial printing materials used in printing material formulations were the HA powder of nanosized grains (NP) with a 3050-nm diameter, air jet milling powder (AP) with a 1030-m diameter, and spherical powder (SP) with a 1050-m diameter. The printing ink viscosity and rheological behavior were investigated. The mechanical properties of different scaffold types were assessed, and the microstructures and morphologies of the printed scaffolds were examined [135]. The results demonstrated that the initial printing substrates could affect the printing quality. Porous scaffolds were successfully printed using the AP and SP inks. However, the NP printing inks showed significant shrinkage, making them unsuitable for bioceramic 3DP. The printing ink formulation affected the macroporous and microporous ceramic structures and mechanical properties. At 60%, 70%, and 80% porosities, the SP scaffold (SPS) demonstrated maximum compressive strengths of 5.5, 3.2, and 0.9 MPa, respectively. The mechanical characteristics of the material drastically deteriorated as the macroporosity increased. The compressive strength of the AP scaffold was marginally greater than that of SPS specimens with identical porosities [128].

A serious health problem that can arise from bacterial adhesion, development, and eventual biofilm formation at an insertion site is implantable medical device infection and subsequent failure. According to Melo et al. [136], CNs, specifically graphene-based compounds (e.g., GO), become possible antibacterial agents when immobilized and exposed in polymeric matrices. The study mainly aimed to develop 3D fibrous scaffolds that incorporate GO, which is antibacterial and biocompatible. Well-defined PCL and composite PCL/GO fibrous scaffolds with an average fiber size of 100 m were manufactured through wet spinning in conjunction with AM. A 5% GO concentration was sufficient for fabricating GO sheets on the composite fiber surface. A time-dependent bactericidal effect of GO and an increase in the death rate from 20% in the neat PCL scaffolds to nearly 80% in the composite scaffolds with 7.5% GO were discovered after the antimicrobial properties of PCL and the composite PCL/GO 3D-organized fibrous scaffolds were evaluated for the first time. According to an in vitro biocompatibility evaluation, human fibroblasts adhered to, disseminated into, and colonized the PCL and composite PCL/GO scaffolds during a 14-day culture period. Consequently, the proposed GO-containing fibrous scaffolds encouraged bacterial death while allowing human cells to function. These characteristics demonstrated how GO inclusion in polymer fiber scaffolds can be used for antibacterial medical implants [136]. The dental applications of 3D printing are common. Yamada et al. [137] developed a dental prosthesis using silver compounds to exert an antibacterial effect on the environment and primarily reduce caries. Sa et al. investigated silver compounds for developing dental restorations with superior antibacterial effects [131].

3D printing is also used for wound treatment. By minimizing tissue swelling and encouraging hemostasis, the use of a wound dressing aids in rapid healing by isolating the injury and its surroundings. Topical agents are used in conjunction with wound dressings to accelerate wound healing and treat any local infections that may have developed by reducing their duration [137,138].

A 3D-printed CS–pectin biopolymeric hydrogel wound dressing containing lidocaine hydrochloride was investigated [139]. These hydrogels were created by cross-linking CS and pectin, and scaffolds were 3D-printed through extrusion-based 3D printing. The wound dressing’s skin adherence, flexibility, and physical integrity were excellent. A constant drug release was also observed, supporting the utility of 3D printing for applications such as wound dressing. Muwaffak et al. used 3D scanning to create a 3D model that could be used to alter the size and contour of a wound dressing [129]. It was created to extrude PCL pellets mixed with various zinc, copper, and silver metal particle loadings. The antibacterial effectiveness of the designed wound dressing against the common *S. aureus* strain revealed that silver and copper wound dressings had the strongest antibacterial effects. Because of this simple, customizable 3D printing method, the development of wound dressings with enhanced antibacterial properties holds great potential [131].

## 8. Conclusions

The demand for and application of tissue engineering, medical devices and tools, and regenerative medicine are increasing, and supply shortages are occurring. AM offers advantages for manufacturing complex medical devices with high precision and low cost. The integrity of medical tools and equipment has been affected by increases in nosocomial infections, biofilm-associated and device-related illnesses, and death counts associated with these diseases. Antimicrobial materials are a promising alternative for combating this situation. This review discussed natural antimicrobial materials, surface treatment of materials using antimicrobial agents, and incorporating antimicrobial agents into biomaterials for fabricating 3D-printed products, highlighting their methods and applications. The antimicrobial mechanisms of different materials have been detailed, and examples of commonly used antimicrobial materials in bioengineering have been provided. Finally, a short overview of their antiviral properties and biomedical applications has been presented.

## Figures and Tables

**Table 1:** Main approaches for designing antibacterial coatings and their features.

Coating	Features
Antimicrobial agent release	Release-based coatings kill both adhered and adjacent bacteria by releasing the loaded antibacterial agents through diffusion into an aqueous medium and erosion/degradation of covalent compounds. The antibacterial activity, which only occurs where needed, minimizes the development of antibacterial resistance [14]. However, these coatings act only temporarily because they have a limited reservoir of antibacterial agents [11].
Contact killing	Contact-killing coatings solve the problem of the limited reservoir of antibacterial agents. Antimicrobial activity is exerted by antibacterial compounds covalently attached to the biomaterial surface through flexible, hydrophobic polymeric chains. This is attributed to the cell membrane disruption of the adhered bacteria by the attached compounds, which reached the microbial envelope due to the long tethering chains, resulting in microbe death [11].
Antiadhesive/bacteria-killing	Antiadhesion coatings, with the help of non-cytotoxic mechanisms, prevent the first step of biofilm formation (i.e., bacterial adhesion to the biomaterial surface, which involves species-specific bacterial adhesion proteins). Molecule immobilization on the surface, which resists the adhesion of proteins like PEG and zwitter ions, is the standard approach for the fabrication of antiadhesive coatings, despite the stability issues, which have shown great antiadhesion results in vitro [11]. With chemistry, the topography of the biomaterial surface (medical device/implant) could be changed to prevent microbial attachment [4,20].

**Table 2:** Summary of materials commonly employed as antibacterial agents, their modes of action and features, and factors affecting their antimicrobial activities.

Material Type	Proposed Action Mode	Features of the Antimicrobial Agent	Factors Affecting the Antimicrobial Activity	References
Silver	Metallic silver is chemically inert. Interaction with water generates silver ions and their biocidal compounds. Interaction with the microbial cell wall leads to cell wall modification; and its ultimate death.	Toxic to bacteria, viruses, and fungi; effective against gram-positive and gram-negative bacteria; and not toxic to humans or mammalian cells.	Chemically inert, effective microbial activity when in the solution	[3,4,5,39,40]
Silver nanoparticles	Ion release; damage to the microbial membrane and the disruption of the cellular functions; binding to the thiol and amine groups; and photocatalytic ability inducing reactive oxygen species (ROS)	They are also effective against drug-resistant strains.	Size and shape of the material	
Zinc	Microbial membrane destabilization due to the direct contact between zinc and membrane; interaction with nucleic acid and inactivation of the enzymes associated with the respiratory system	Effective against bacterial and fungal strains as well as viruses	Depending on the concentration and contact duration	[41,42,43]
Zinc oxide	ROS generation; destabilization of the membrane due to the direct interaction between the microbial membranes, zinc oxide particles, and the cell wall; the zinc ions released have an intrinsic antimicrobial property	Efficient in combating both gram-positive and gram-negative bacteria as well as viruses; high antimicrobial activity, low toxicity, and easy clearance	Concentration, size, and surface area of ZnO particles	
Copper	Cell membrane breakdown, intracellular biochemical process modification, and DNA damage initiation	Effective against gram-positive and gram-negative bacteria; drug-resistant strains and antifungal activity	pH, carbon sources, and temperature	[4,40,44]
Copper oxide nanoparticles	ROS generation; nanoparticles that cross the bacterial cell membrane and harm the microorganism’s essential enzymes	Effective against gram-positive and gram-negative bacteria; high stability; antifungal activity; and drug-resistant strains; cheaper	Particle size and concentration	
Chitosan	Bacterial biofilm splits might be caused by the interaction between the positively charged chitosan molecules and negatively charged microbial cell walls, which leads to the leaking of the resultant proteins and different other cell elements, resulting in microbial death	Effective against algae, bacteria, fungi, sporulation, and spore germination	Several cationized ions on the polymeric surface; the polarity of the microbial surface: pH, temperature, time, kind of microorganism, chitosan’s molecular weight, hydrophilic and hydrophobic attributes, physical state, its ability to chelate substances, ionic strength, and its concentration are all factors.	[45,46,47]
TIO_2_	Light absorption, electron/hole generation, ROS-mediated organic material oxidation	Effective against gram-positive and gram-negative bacteria, viral species, parasites, and bacillus spores; photocatalytic property	Temperature and degree of the polymorphic reaction	[4,40,48]
TiO_2_ Nanoparticle	Oxidative stress via ROS generation; lipid peroxidation that causes enhanced membrane fluidity and disrupts cell integrity	High stability, suitable photocatalytic characteristics, and potent antifungal activity against fluconazole-resistant microbes	Crystal structure, shape, and size	
Hydrox apatite	Metals and metal nanoparticles incorporated into hydroxyapatite are responsible for the respective antimicrobial activity in hydroxyapatite-based systems.	Effective against bacteria, viruses, eukaryotic organisms, and fungi Exhibit high biocompatibility and mechanical properties, such as high bond strength and elastic modulus, for biomedical applications	Chemical composition of ceramics and the degradation condition around ceramics	[49,50,51]
Carbon nanotubes	The membrane is disrupted because of membrane oxidation due to the electrostatic interactions between the microorganisms’ outer surface and carbon nanotubes (CNT). Reactive oxygen species can either inhibit the bacteria’s biological molecules or damage the DNA. Any contaminant added to the CNT structure during manufacture may enhance its antibacterial properties.	Efficient against gram-positive and gram-negative bacteria Carbon nanotubes are divided into three groups based on their structural characteristics: single-walled carbon nanotubes (SWCNTs), double-walled carbon nanotubes, and multi-walled carbon nanotubes (MWCNTs). Each structure is composed of a single, double, or several layers of graphene cylinders. SWCNTs have better antimicrobial activity in comparison to MWCNT’s	Diameter length, presence of a residual catalyst, coating, electronic structure; functional group, and surface chemistry of the carbon nanotubes	[52,53]
Graphene	Deep insertion and disruption of the cell membrane; destructive removal of phospholipids from the lipid membranes; ROS-induced oxidative stress causes substantial damage to lipids and proteins (cellular constituents); because graphene interferes with the unique bacterial processes, oxidative stress is produced without ROS through the oxidation and destruction of significant biological structures, and taking the microbial cell out of its surroundings and isolating it	Efficient against gram-positive and gram-negative bacteria and fungi. Graphene and graphene-based compounds exhibit exceptional properties, including large surface areas and distinctive, thermal, electrical, and physicomechanical characteristics, which make them effective antimicrobials. In contrast to carbon-based materials, graphene, and its compounds (e.g., graphene oxide) are quick and easy to make, and inexpensive. At low doses, GO is barely harmful to mammalian cells	Temperature, time, pH, concentration, length, and surface area of graphene and graphene-based compounds	[54,55,56]
Diamond-like carbon	Microbes suffer from direct physical harm because of severe membrane disruption and the release of microbial intercellular metabolites; the DLC films demonstrate an anti-biofouling/antibacterial mpact based on the surface profile; each DLC film has a unique property that depends on the circumstances at the time of the DLC structure production; and the sp3/sp2 ratio is crucial to the biological functions of the DLC structures.	Because of properties like excellent biocompatibility, chemical inertness, and superior mechanical properties, DLC is an excellent candidate as an antimicrobial material used in biomedical applications.	Hydrophobicity, the dispersive part of the surface energy, and smoothness	[56,57]

**Table 3:** Mechanisms of antimicrobial structures for repelling or eliminating microorganisms.

Drug-Loaded Polymers	Polymeric Hydrogels	Surface-Bound Polymers
Micelles, vesicles, nanoparticles, and dendritic structures. Drugs or other biocidal substances are delivered or released [87,88,89]	Gel-like microstructures. Using medicines or biocides to get rid of microbes [82,90,91]	Surface-bound polymer—structures have many different structures, including brushes, rods, fibers, worms, and spherical nanoparticles. Neutral polymer-based surfaces (steric-repellent properties) Anionic polymer-based surfaces (electrostatic repellent properties). Ultra hydrophobic— (polymer-based surfaces made of low energy). Contact-killing—surfaces (cationic, employment of biocidal compounds) Biocide-releasing surfaces (biocide discharge) Stimuli-responsive surfaces (temperature, pH, etc.) Adaptive bacterial surfaces [89,92,93]

## Data Availability

Data are contained within the article.
